# Controlled Sr(ii) ion release from *in situ* crosslinking electroactive hydrogels with potential for the treatment of infections

**DOI:** 10.1039/d3ra07061a

**Published:** 2024-01-31

**Authors:** Melike Fırlak Demirkan, Dilek Öztürk, Zeynep Sude Çifçibaşı, Fatma Ertan, John George Hardy, Aslı Nurşeval Oyunlu, Hakan Darıcı

**Affiliations:** a Department of Chemistry, Gebze Technical University Gebze Kocaeli 41400 Turkey mfirlak@gtu.edu.tr; b Department of Chemistry, Lancaster University Lancaster LA1 4YB UK; c HD Bioink Biotechnology Corp. İstanbul Turkey; d 3D Bioprinting Design & Prototyping R&D Center, Istinye University Istanbul Turkey; e Faculty of Medicine, Dept. of Histology & Embryology, Istinye University Istanbul Turkey; f Stem Cell, and Tissue Engineering R&D Center, Istinye University Istanbul Turkey

## Abstract

The development of electrochemical stimuli-responsive drug delivery systems is of both academic and industrial interest due to the ease with which it is possible to trigger payload release, providing drug delivery in a controllable manner. Herein, the preparation of *in situ* forming hydrogels including electroactive polypyrrole nanoparticles (PPy-NPs) where Sr^2+^ ions are electrochemically loaded for electrically triggered release of Sr^2+^ ions is reported. The hydrogels were characterized by a variety of techniques including Fourier-transform infrared (FTIR) spectroscopy, scanning electron microscopy (SEM), energy dispersive X-ray spectroscopy (EDX), thermogravimetric analysis (TGA), X-ray diffraction (XRD), cyclic voltammetry (CV), *etc.* The cytocompatibility towards human mesenchymal stem cells (MSCs) and fibroblasts were also studied. The Sr^2+^ ion loaded PEC-ALD/CS/PPy-NPs hydrogel showed no significant cytotoxicity towards human mesenchymal stem cells (MSCs) and fibroblasts. Sr^2+^ ions were electrochemically loaded and released from the electroactive hydrogels, and the application of an electrical stimulus enhanced the release of Sr^2+^ ions from gels by *ca.* 2–4 fold relative to the passive release control experiment. The antibacterial activity of Sr^2+^ ions against *E. coli* and *S. aureus* was demonstrated *in vitro*. Although these prototypical examples of Sr^2+^ loaded electroactive gels don't release sufficient Sr^2+^ ions to show antibacterial activity against *E. coli* and *S. aureus*, we believe future iterations with optimised physical properties of the gels will be capable of doing so.

## Abstract 2

The first example of Sr^2+^ ion release from *in situ* crosslinking electroactive hydrogels in response to the application of electricity.

## Graphical abstract

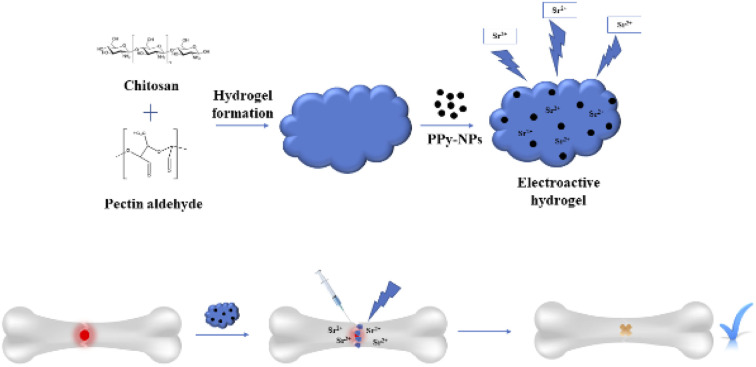

## Introduction

Controlled release technology generates economic, health and societal impacts.^1,2^ Controlled release technology can ensure high efficacy of treatment,^3^ at low cost,^4^ with reduced risks of toxicity and other side effects of the drug,^5^ providing stable levels of drugs in blood and plasma and greater patient comfort,^6^ potentially with delivery of precise quantities of drugs at specific locations and times, hence they can be tailored to each person's needs.^7^ There are various external stimuli being explored including light, pH, temperature, electricity, enzymes and magnetic fields,^8^ that can potentially be triggered externally from the body. Each type of stimuli has its own advantages; in the case of electrical triggers including switchable ON–OFF release,^9^ the magnitude of current (and concomitantly amount of drug) can be controlled precisely.^10^

Conducting polymers (CPs) are a family of polymers with extended π-bonding that can confer electrical properties close to semiconductors/metals, and potentially interesting electrochemical and optical properties,^11^ useful for a variety of technologies/industries. CPs (*e.g.*, polyaniline (PANI), polypyrrole (PPy), poly(3,4-ethylenedioxythiophene) (PEDOT), and their derivatives) can be synthesised by a variety of methods.^12–14^

There is a market need for biocompatible materials capable of well controlled drug delivery,^15^ hydrogels are one class of materials used for drug delivery,^16^ tissue engineering^17^ and biosensors.^18^*In situ* forming hydrogels can be used in the emerging field of patient-specific treatments (*e.g.* drug delivery, cell therapy, *etc.*).^19^ Biopolymers (*e.g.*, chitosan, dextran, gelatin, hyaluronic acid, pectin, *etc.*) can be used to produce *in situ* forming hydrogels for drug delivery studies,^20^ often employing Schiff base reactions.^19^

Metal ions (*e.g.*, Ca^2+^, Cu^2+^, Fe^2+^/Fe^3+^, K^+^, Mg^2+^, Na^+^, Zn^2+^) play a variety of important roles in biology,^21–23^ motivating the investigation of therapeutic systems incorporating metal ions.^24–32^ Elegant examples of ion delivery systems include organic electronic ion pumps which have been used to deliver a variety of metal ions including K^+^,^33^ Ca^2+^ (ref. 34) and are the subject of a review.^35^

Sr^2+^ ions play a significant role during new bone formation by stimulating osteoblasts^36^ and simultaneous inhibition of bone resorption by suspending the function of osteoclasts.^37^ For this reason, several formulations have been used to treat bone diseases and other bone related conditions.^38^ Some Sr containing minerals display antibacterial activity, however, the effects of Sr^2+^ are highly related to its concentration, with excess amounts of Sr^2+^ causing osteonecrosis,^39^ therefore, the amount of Sr^2+^ ions released should be well controlled.^40^

Furthermore, we note that these nanostructured electroactive biomaterials have potential dual use for delivery of bioactive species,^41^ and moreover, regenerative bone formation triggered by electrical stimulation of cells.^42–45^ Consequently, we believe that controlled release of Sr^2+^ ions from biomaterials (*e.g.*, *in situ* crosslinking electroactive hydrogels) have great promise for the treatment of bone diseases/trauma. Additionally, such biomaterials may be easily repurposed for the delivery of other divalent cations such as Zn^2+^ and Ca^2+^ ions.^46^ By using electroactive hydrogels, a portion of the market demand for dosage-controlled release of divalent ions may be met.

Here we report the preparation of *in situ* forming electroactive composite hydrogels formed by Schiff base formation between aldehydes displayed on an oxidized derivative of pectin (PEC-ALD) and amines displayed on chitosan (CS) including PPy-NPs, and the characterisation of their physicochemical properties. Sr^2+^ ions were loaded electrochemically into the hydrogels, and the electrically triggered release of Sr^2+^ ions was investigated. The cytocompatibility of the hydrogels towards Human Mesenchymal Stem Cells (MSCs) and fibroblasts were studied, as was the antibacterial activity of the Sr^2+^ ions released from the gels towards *E. coli* and *S. aureus*.

## Experimental

### Materials

Unless otherwise stated, all chemicals and consumables were purchased from either Sigma Aldrich or Acros Organics, of analytical grade, and used as supplied.

### Synthesis of pectin displaying aldehydes

In order to introduce aldehyde groups, pectin from citrus peel (poly-d-galacturonic acid methyl ester, 21 000–70 000 Da) was oxidized by adaptation of the literature.^19,47^ 1.0 g of pectin from citrus peel was dissolved in 100 mL ultrapure (Millipore) water. Then, the oxidant agent sodium periodate (0.535 g, 2.5 mM) was added, and the reaction was stirred for 24 h at room temperature in the dark. Finally, ethylene glycol (140 μL, 2.5 mM) was added to the reaction mixture to eliminate the unreacted periodate and stirred the reaction for 1 h at room temperature in the dark. Low molecular weight contaminants were removed by dialysis using ultrapure water in dialysis tubing cellulose membrane (MWCO 3500). Dialysis was performed for four days with water exchange every 2 hours during the daytime. Then PEC-ALD was freeze-dried. After freeze-drying PEC-ALD was obtained with an 80.54% yield.

### Preparation of chitosan stock solution

Commercial chitosan with medium molecular weight (190 000–310 000 Da, 75–85% deacetylated) was used as –NH_2_ source for the *in situ* crosslinking hydrogel. A 2 w% chitosan (CS) stock solution was prepared adding the CS powder into a 1 vol% acetic acid aqueous solution at ambient temperature and stirred until CS was completely dissolved. After complete dissolution, the pH of the solution was adjusted to 5.5 by drop-wise addition of 1 M sodium hydroxide. The formulation was then stirred for 24 h.^48^

### Preparation of polypyrrole nanoparticles (PPy-NPs)

PPy-NPs were prepared by adaptation of the literature.^49^ Briefly, sodium dodecyl sulphate (SDS, 0.86 g) was dissolved in 30 mL distilled water by stirring for 30 minutes. Then, 1 g of pyrrole was added to the solution drop by drop and 5.55 g of FeCl_3_ was dissolved in 5 mL distilled water which was added dropwise to the mixture. The polymerization process was carried out for 3 hours at room temperature. The black PPy precipitate was filtered off and washed with water and ethanol until clear wash liquid obtained. The black PPy-NPs powder was dried in a vacuum oven at 40 °C overnight. After drying PPy-NPs were obtained with 88.44% yield.

### Preparation of *in situ* crosslinking hydrogel (PEC-ALD/CS)

For preparation of *in situ* crosslinking hydrogels, 1 w% solutions of PEC-ALD in phosphate buffered saline (PBS, pH 7.4) and 2 w% solution of CS in acidified water (pH 5.5) were used. By mixing the stock solutions at a volume ratio of 1 : 2 for PEC-ALD/CS and stirring for 2 minutes crosslinking translucent gels were obtained.

### Preparation of electroactive *in situ* crosslinking hydrogel (PEC-ALD/CS/PPy-NPs)

The stock solutions of CS and PEC-ALD were prepared as described above. Then, four different percentages of PPy-NPs were added to the composition of PEC-ALD/CS hydrogel. The percentage of the PPy-NPs was calculated according to the total mass of the polysaccharides and presented in Table 1. The pre-weighed amount of PPy-NPs was transferred into a container and the stock solutions of the polysaccharides at a volume ratio of 1 : 2 for PEC-ALD : CS were added into the container and the mixture was stirred for 2 minutes yielding homogenous black electroactive gels.

**Table 1:** PPy-NPs content of different electroactive *in situ* crosslinking hydrogels

Formulation	PPy-NPs: polysaccharide percentage (w/w)
PEC-ALD/CS	0
PEC-ALD/CS/PPy-NPs-1	2.5
PEC-ALD/CS/PPy-NPs-2	5.0
PEC-ALD/CS/PPy-NPs-3	7.5
PEC-ALD/CS/PPy-NPs-4	10.0

### Fourier transform infrared spectroscopy (FTIR)

Infrared spectroscopy was carried out on a PerkinElmer Spectrum 100 spectrometer. Spectra were recorded as an average of 16 scans in ATR mode at room temperature, with a 1 cm^−1^ resolution.

### *In vitro* swelling studies

The hydrogels were dried in a vacuum oven at 40 °C overnight and dry hydrogels weighed and immersed in PBS solutions (pH 7.4) at 37 °C for 24 hours. Then, the swollen hydrogels were removed and immediately weighed after the excess water on their surfaces was blotted away with filter paper, until the weight of hydrogels reached an equilibrium value. The swelling percentage (SP) was calculated as follows: SP (%) = ((*W*_24_ − *W*_0_)/*W*_0_) × 100, where *W*_24_ and *W*_0_ are the weights of the hydrogels in the swollen state and dry state, respectively.

### X-ray diffraction (XRD)

X-ray diffraction patterns of the samples were obtained using a Bruker powder diffractometer (D8 Advance, Bruker, Germany). Analyses were carried out with Cu Kα radiation (0.15406 nm) at accelerating current of 40 mA and voltage of 45 kV and data were collected with a scan range of 2*θ* = 10°–80° for determination of crystallographic properties for the samples.

### Particle size distribution analysis for PPy-NPs

Particle size distribution of PPy-NPs was analysed using a Mastersizer 2000 analyser (Malvern Panalytical Ltd, United Kingdom).

### Scanning electron microscopy-energy dispersive spectroscopy (SEM-EDS)

The surface morphologies of the PEC-ALD/CS and PEC-ALD/CS/PPy-NPs *in situ* crosslinking hydrogels were investigated by SEM-EDS (Philips XL 30 S-FEG, the Netherlands) equipped with an EDAX detector (Ametek, USA, Apollo X) for energy dispersive X-ray analysis.

### Thermal analysis

Thermal gravimetric analysis (TGA) and differential scanning calorimetry analysis (DSC) of the PEC-ALD/CS and PEC-ALD/CS/PPy-NPs *in situ* crosslinking hydrogels were performed using a TGA/SDTA851e model TGA instrument (Mettler Toledo, Switzerland) and DSC822e model DSC instrument (Mettler Toledo, Switzerland), respectively. Temperatures observed were from 25 to 700 °C with a heating rate of 10 °C min^−1^ under an argon flow (50 mL min^−1^) and −40 to 300 °C with a heating rate of 10 °C min^−1^ for TGA and DSC, respectively.

### Cyclic voltammetry (CV)

Cyclic voltammetry measurements were performed using DropSens μSTAT 400 potentiostat connected to a personal computer using the Dropview 8400 software. For CV measurements, a three-electrode system was used with an Ag/AgCl reference electrode, a platinum-wire counter electrode, and a glassy carbon electrode with the PEC-ALD/CS/PPy-NPs *in situ* crosslinking hydrogel working electrode. The electrodes were in a biomedically relevant buffer (4 mL of phosphate-buffered saline [PBS] at pH 7.4). For CV measurements, the potential was swept between −1.0 V and +1.0 V *vs.* the Ag/AgCl electrode at a scan rate of 0.05 V s^−1^.

### Electrochemical Sr^2+^ loading studies

Sr^2+^ loading into the PEC-ALD/CS and PEC-ALD/CS/PPy-NPs electroactive hydrogels was carried out electrochemically using a DropSens μSTAT 400 potentiostat connected to a personal computer using the Dropview 8400 software (amperometric technique). A three-electrode system was used with an Ag/AgCl reference electrode, a platinum-wire counter electrode, and a glassy carbon electrode with the electroactive *in situ* crosslinking hydrogel (PEC-ALD/CS/PPy-NPs) working electrode in an aqueous solution of Sr^2+^ ion (100 mM SrCl_2_·6H_2_0). Prior to electrical stimulation, there was a quiet time of 20 s at a potential of −0.1 V, after which the PEC-ALD/CS/PPy-NPs *in situ* crosslinking hydrogel working electrodes were stimulated for 30 minutes with a potential of −0.6 V. The concentrations of Sr^2+^ solutions before and after loading were determined using inductively coupled plasma optical emission spectroscopy (ICP-OES) (PerkinElmer, USA, Optima DV).

### Electrochemical Sr^2+^ release studies

The electrochemically triggered release of Sr^2+^ ions from the hydrogels was achieved using a DropSens μSTAT 400 potentiostat connected to a personal computer using the Dropview 8400 software (amperometric technique), and a three-electrode system (described above) in a biomedically relevant buffer (4 mL of PBS at pH 7.4). The data are reported as cumulative release as a percentage of the total mass of the Sr^2+^ ions in the hydrogels (hydrogels were individually weighed and the loaded concentration of Sr^2+^ ions were individually determined prior to Sr^2+^ ion release experiments).

### Antibacterial activity studies

The antibacterial activity of the Sr^2+^ ions was tested against Gram-negative (*E. coli* ATCC 8739) and Gram-positive (*S. aureus* ATCC 29213) bacterial strains using the agar-well diffusion method.^50,51^ The bacterium strains were provided by the Department of Microbiology, Gebze Technical University. *E. coli* and *S. aureus* were cultured in Luria–Bertani (LB) and Nutrient agar (NA) at 37 °C (180 rpm), respectively.^52^ The bacterial concentrations were adjusted to approximately 0.1 optical density at 600 nm. Subsequently, nutrient and LB agar plates were inoculated uniformly by spreading 100 μL cell suspension of bacteria with a sterile dragalski spatula all over the plate in one direction. 100 mg Sr^2+^ ions were dissolved in 1 mL of physiological solution (0.9% NaCl). Four holes with a diameter of 5 mm were punched with a sterile tip and 10–100 μL of strontium solution was introduced into the wells. For this test, chloramphenicol discs (30 μg) were used as a positive control. Agar plates were incubated for 24 h, at 37 °C. The antibacterial effect of Sr^2+^ ions solution diffused into the agar medium was determined by measuring the zone of inhibition in mm. In addition, the electrically triggered released Sr^2+^ ions solutions were also separately introduced into the wells and antibacterial activity of the released Sr^2+^ ions concentration was investigated. The concentration of the released Sr^2+^ ions was determined by using ICP-OES. The test was performed with three replicates for each Sr^2+^ ions concentration.

### CCK8 cell viability assay

ATCC PSC-500-010 Human Mesenchymal Stem Cells (MSCs) and ATCC PCS-201-012 fibroblasts were cultured with DMEM/F12 media with 10% fetal bovine serum and 1% penicillin–streptomycin addition in a 37 °C incubator with 5% CO_2_ until cells reach enough number. The cells to be treated were seeded at 0.01 × 10^6^ cells per well in a 96-well plate and cultured overnight for adhesion.

Hydrogels were weighed and autoclaved at 121 °C for 15 minutes for sterilization.^53^ Sterile gel beads were divided into equal pieces then placed on top of the cells in a laminar flow hood. At least five samples were used for each weight while no gels were added to control group cells. Cells were cultured with or without hydrogels for 48 hours to observe any harmful effect. Since the hydrogels are black in color which may cause interference in spectroscopy, they were removed along with media. Culture media was renewed with 190 μL of DMEM/F12 with addition of 10 μL CCK8 solution for each well. Cells were kept in the incubator for another 2 hours. Absorption measurements were taken using the spectrophotometry (SPECTROstar) device and SPECTROstar nano application. Graphics and standard deviations (SDs) were processed in excel.

## Results and discussion

### Synthesis of pectin displaying aldehydes

The chemically crosslinked hydrogels were synthesised *via* Schiff base reaction between aldehyde displaying PEC-ALD and amine displaying chitosan. The Schiff base reaction between these functional groups rapidly occurs and enables formation of the hydrogels in seconds to minutes.^19,54–57^ In order to obtain aldehyde displaying pectin (PEC-ALD), pectin was oxidized using sodium periodate.^19^

### Preparation of *in situ* crosslinking hydrogel (PEC-ALD/CS) and electroactive *in situ* crosslinking hydrogel (PEC-ALD/CS/PPy-NPs)

**Fig. 1:**
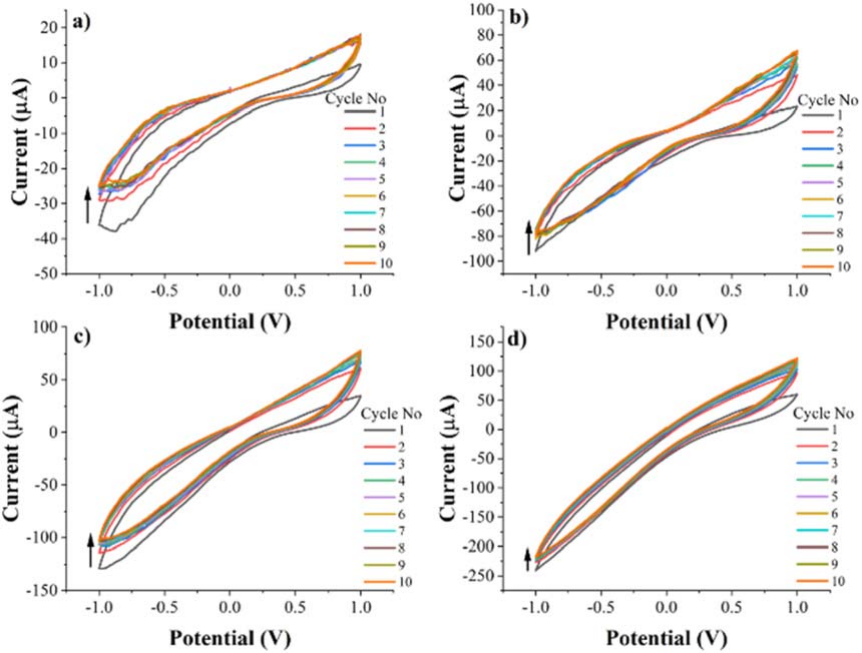
Cyclic voltammetry (CV) data of electroactive hydrogel with (a) 2.5% PPy-NPs (b) 5% PPy-NPs (c) 7.5% PPy-NPs and (d) 10% PPy-NPs in phosphate-buffered saline (PBS; pH = 7.4) at a scan rate of 50 mV s^−1^ (10 cycles).

A 1 w% solution of PEC-ALD in phosphate buffered saline (PBS, pH 7.4) was prepared. A 2 w% CS solution was prepared in acidified water and its pH was adjusted to pH 5.5. The stock solutions described above were mixed at a volume ratio of 1 : 2 for PEC-ALD : CS, crosslinking occurred within seconds and complete gelation was obtained after mixing the components for two minutes, yielding either translucent PEC-ALD/CS electroactive gels or black and PEC-ALD/CS/PPy-NPs electroactive gels by the addition of PPy-NPs. The electroactive behaviour of the PEC-ALD/CS/PPy-NPs electroactive gels including different amount of PPy-NPs was investigated by CV technique. The conductivity of the hydrogels was observed to increase as the amount of PPy-NPs increases (Fig. 1). 5% PPy-NPs (PEC-ALD/CS/PPy-NPs-2) was decided to use for further studies, since the increased amount of PPy-NPs causes deterioration in the physical structure of the hydrogel.

### Fourier transform infrared spectroscopy

**Fig. 2:**
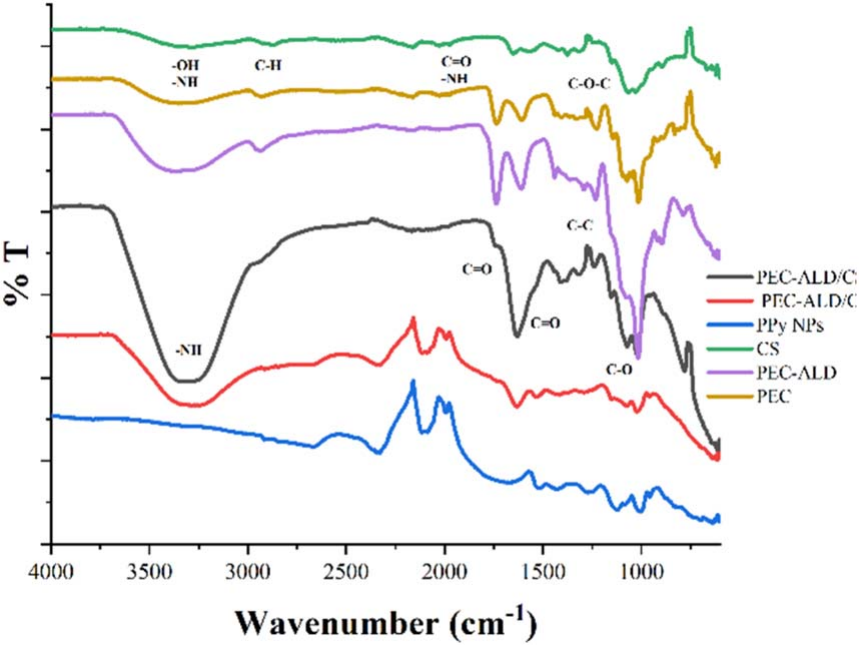
FTIR spectra of CS, PEC-ALD, PPy-NPs, PEC-ALD/CS hydrogel and PEC-ALD/CS/PPy-NPs hydrogel.

The FTIR spectra of CS, PEC, PEC-ALD, PPy-NPs and corresponding hydrogels are displayed in Fig. 2. The characteristic O–H and C–H stretching vibration peaks were observed at 3370 and 2932 cm^−1^. The peaks at 1733 cm^−1^ and 1610 cm^−1^ were attributed to the carboxymethyl (−COOCH_3_) and C=O peaks of pectin. The peak at 1017 cm^−1^ was attributed the C–O stretch of the secondary alcohol groups.^58^ The FTIR spectrum of PEC-ALD showed that the intensity of peaks increased after oxidizing pectin yielding PEC-ALD.

The FTIR spectrum of CS showed a broad band at around 3353–3291 cm^−1^ which was attributed to O–H and N–H stretching vibrations. A small peak at 2870 cm^−1^ corresponded C–H symmetric and asymmetric stretching vibrations. These bands are characteristic bands for polysaccharides and found in other polysaccharides such as glucans, xylan, *etc.*, too.^59^ The peak at 1648 cm^−1^ related to C=O group of amide I and bending vibrations of the N–H (*N*-acetylated residues, amide II band) absorption band was observed at 1562 cm^−1^. The absorption band at 1150 cm^−1^ can be attributed to asymmetric stretching of the C–O–C bridge. The skeletal C–O stretching peaks were observed at 1065–1027 cm^−1^.^60^ The FTIR spectrum of the PEC-ALD/CS hydrogel which was obtained by mixing PEC-ALD and CS exhibited a weak shoulder and sharp peaks at 3330 cm^−1^ is attributed to amine groups on CS, 1730 cm^−1^ and 1630 cm^−1^ are attributed to C=O stretching of carboxylic acid on both PEC-ALD and CS. The PEC-ALD/CS/PPy-NPs hydrogel was prepared by mixing PEC-ALD, CS and PPy-NPs and the FTIR spectrum of the hydrogel showed characteristic vibration peaks at 1529–1431 cm^−1^ that can be attributed to the pyrrole ring stretching of the PPy-NPs. The peak at 1280 cm^−1^ due to the C–C in-ring stretching and C–N deformation mode.^61^ The FTIR spectrum of the PEC-ALD/CS/PPy-NPs hydrogel confirmed that PPy-NPs were well mixed with the hydrogel.

### *In vitro* swelling studies

The swelling behaviour of the PEC-ALD/CS and PEC-ALD/CS/PPy-NPs gels in PBS was investigated. The swelling percentage for PEC-ALD/CS hydrogel was found to be 208 ± 10, whereas that of PEC-ALD/CS/PPy-NPs hydrogel was found to be 525 ± 7. The addition of PPy-NPs to the gel formulation resulted in higher swelling percentages than the PEC-ALD/CS hydrogel formulation, likely due to the presence of PPy-NPs that diminishes the number of Schiff base crosslinks that form, thereby increasing the pore sizes within the hydrogel.^19,54–56^

### X-ray diffraction

**Fig. 3:**
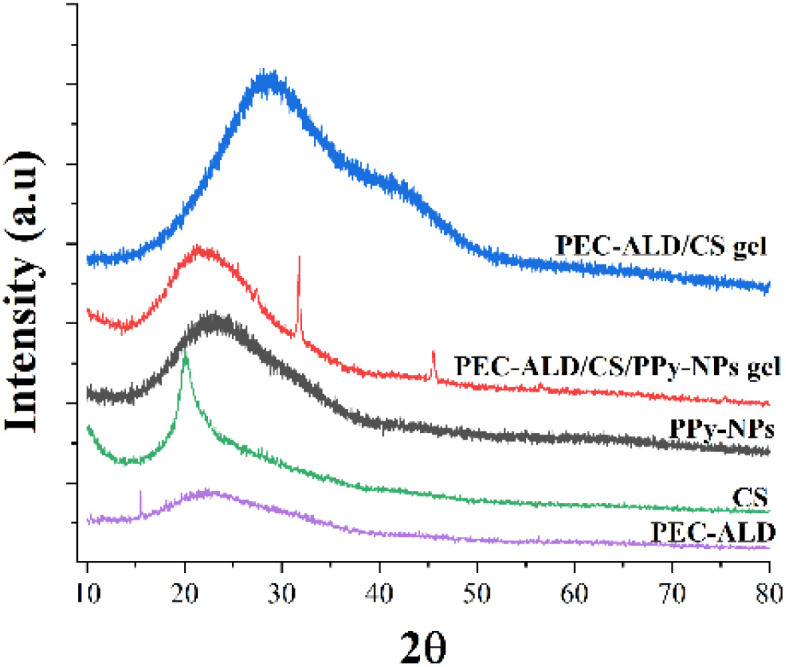
XRD patterns of chitosan, PEC-ALD, PPy-NP, PEC-ALD/CS and PEC-ALD/CS/PPy-NPs gels.

XRD was employed to provide more information on the individual components of the gels, and PEC-ALD/CS and the PEC-ALD/CS/PPy-NPs gels' structures. XRD patterns of CS, PEC-ALD, PPy-NPs, PEC-ALD/CS gel, and PEC-ALD/CS/PPy-NPs gel are shown in Fig. 3. The XRD pattern of CS is in agreement with literature showing a broad peak with a sharp top at 2*θ* = 20° which is a clear evidence of a semi-crystalline structure.^19^ A sharp peak at 2*θ* = 15.57° and a broad peak centred around 2*θ* = 25° of PEC-ALD were observed indicating PEC-ALD has mostly amorphous structure with partial crystallinity.^19,62,63^ The XRD pattern of the PPy-NPs showed a broad peak centred at 2*θ* value *ca.* 26° confirming amorphous nature of PPy-NPs.^63,64^ The XRD pattern of PEC-ALD/CS gel showed a very broad peak at around 2*θ* = 30° with a shoulder centred around at 2*θ* = 40° that indicates PEC-ALD/CS gel has an amorphous structure,^19^ whereas the pattern of the PEC-ALD/CS/PPy-NPs gel showed a broad peak at around 2*θ* = 22° and four sharp peaks at 2*θ* = 31.78, 45.57, 58.59 and 75.49° correspond to NaCl (2*θ* = 31.78, 45.57 and 58.59)^65^ and KCl (2*θ* = 31.78, 58.59 and 75.49°)^66^ in the gel.

### Particle size distribution analysis for PPy-NPs

The particle size of the nanoparticles is an important parameter underpinning the injectability of the hydrogels including the nanoparticles. In order to understand suitability of the PPy-NPs for *in situ* crosslinking hydrogels and needle gauge necessary, particle size distribution analysis was performed using a Mastersizer. The particle size of the PPy-NPs was found to be 112 ± 29 nm, however a few particles were observed to have agglomerated. The size of the nanoparticles was also confirmed using scanning electron microscopy and it can be seen that particle size is mostly around 110 nm and the diameters of aggregates are around 250 nm which is still injectable *via* needles of a variety of gauge sizes used in surgical procedures.^67–74^

### Scanning electron microscopy (SEM)

**Fig. 4:**
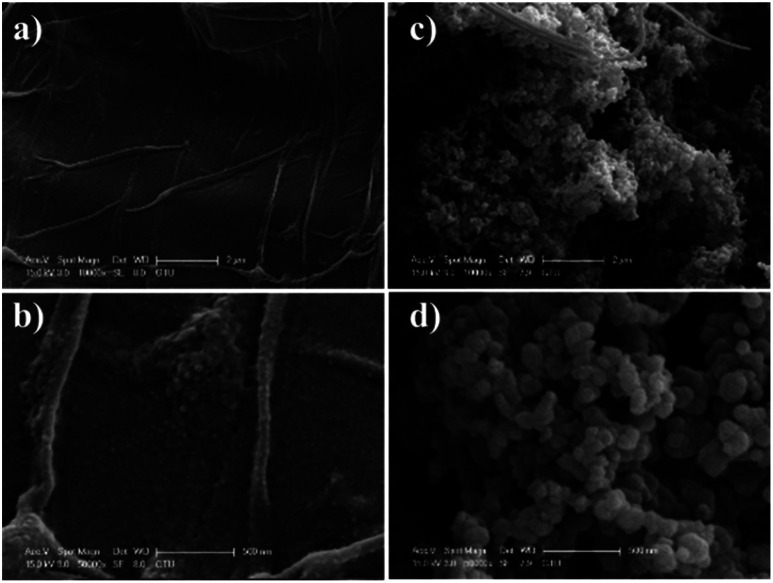
SEM images of PEC-ALD/CS (a and b) and PEC-ALD/CS/PPy-NPs gels (c and d). Scale bars represent 2, 2, 0.5, 0.5 μm in (a)–(d), respectively.

**Fig. 5:**
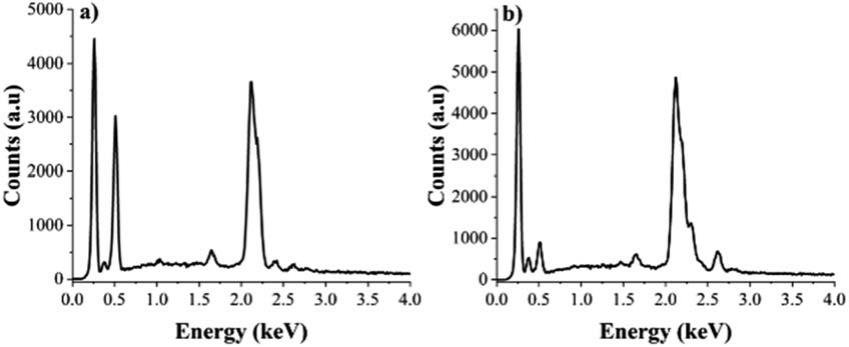
(a) EDS data from a PEC-ALD/CS gel (b) EDS data from a PEC-ALD/CS/PPy-NPs gel.

The surface morphology of the PEC-ALD/CS and PEC-ALD/CS/PPy-NPs gels were investigated using SEM are shown in Fig. 5. As it can be seen from Fig. 4a and b, PEC-ALD/CS gels have smooth surfaces with heterogeneously distributed pores, while PEC-ALD/CS/PPy-NPs gels have larger pores. SEM images of PEC-ALD/CS and PEC-ALD/CS/PPy-NPs gels are in good agreement with the swelling percentage data. On the other hand, as it can be seen in Fig. 4, comparing the PEC-ALD/CS gel with the PEC-ALD/CS/PPy-NPs gel, it is evident the presence of agglomerated PPy-NPs in the electroactive gel. However, the SEM images of PEC-ALD/CS/PPy-NPs gel, show homogeneously distributed aggregates inside the gel matrix. It is also possible to observe the difference of the network structure when polymerized in the absence or presence of the nanoparticles. In Fig. 4c and d, evidence of agglomerated PPy-NPs can be observed. By comparison in the case of PEC-ALD/CS gels, it is possible to observe in Fig. 4a and b, the presence of fibrillar structures in the hydrogel network. Additionally, the particle size of PPy-NPs and agglomerates in the SEM images are in good agreement with particle size distribution data determined using a Mastersizer.

### Energy dispersive spectroscopy (EDS)

The EDS analysis of the PEC-ALD/CS and PEC-ALD/CS/PPy-NPs gels was performed, and results are given in Fig. 5 and Table 2. The elemental analysis results confirm the presence of PPy-NPs in PEC-ALD/CS/PPy-NPs gel as there is a clear increase in the percentages of C and N atoms while the percentage of O atoms decreased (Table 2). Analysis of the EDS data for PEC-ALD/CS and PEC-ALD/CS/PPy-NPs gels (Fig. 5a and b, respectively) showed elemental signals characteristic of carbon (Kα at 0.277 keV), nitrogen (Kα at 0.392 keV) and oxygen (Kα at 0.525 keV) found on CS, PEC-ALD and PPy, respectively.

**Table 2:** Percentage mass of C, O, N atoms in *in situ* crosslinking hydrogels assessed *via* EDS

Element	Composition (%) – PEC-ALD/CS gel	Composition (%) – PEC-ALD/CS/PPy-NPs gel
C	44.43	55.16
N	10.56	23.46
O	45.01	21.38

### Thermogravimetric analysis

**Fig. 6:**
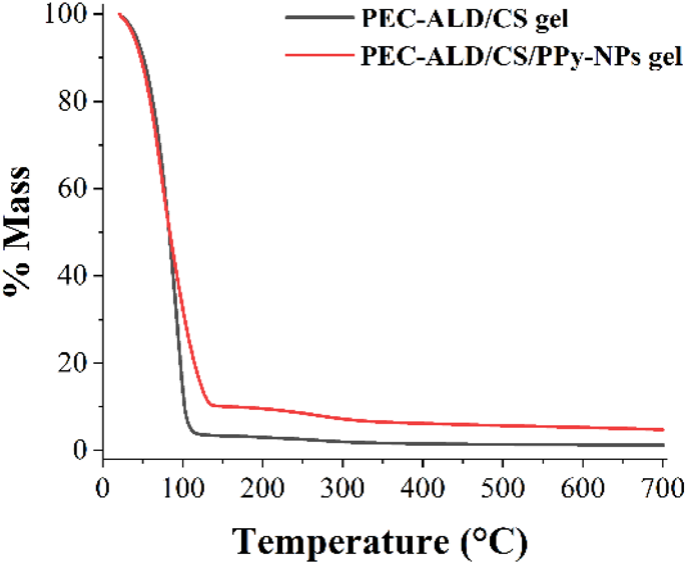
TGA thermograms of the PEC-ALD/CS and PEC-ALD/CS/PPy-NPs hydrogels.

Fig. 6 shows TGA thermograms of the PEC-ALD/CS and PEC-ALD/CS/PPy-NPs hydrogels. Main mass loss due to the evaporation of the water was observed from 20 °C to approximately 100 °C for PEC-ALD/CS gels whereas that of for PEC-ALD/CS/PPy-NPs gels was from 20 °C to approximately 135 °C. All gels showed major weight loss due to evaporation of water. Then the peak at *ca.* 200 °C and *ca.* 220 °C correspond to the mass loss upon degradation of the PEC-ALD/CS and PEC-ALD/CS/PPy-NPs hydrogels, respectively. Pure chitosan shows a distinct weight loss at between 200–300 °C, attributable to the degradation of chitosan chains. Addition of other components into the structure showed that thermal stability of the gel was decreased.^75,76^ PEC-ALD shows two steps degradation, first step occurs at between 150–250 °C and second step occurs at between 250–300 °C.^77^ The thermal stability of the *in situ* crosslinking hydrogels can be attributed to the presence of strong contact between –CHO groups of PEC-ALD and –NH_2_ group of CS that have reacted to form Schiff bases.^77^ The TG analysis of the hydrogels is in a good agreement with the literature and show that addition of the electroactive PPy-NPs to the hydrogel matrix increased the thermal stability of the hydrogels (potentially due to polysaccharide-PPY interactions).

**Fig. 7:**
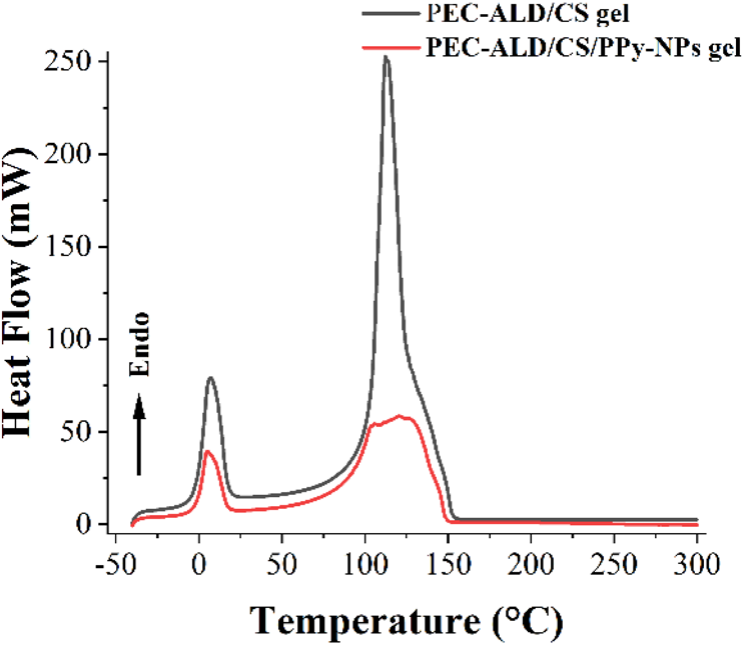
DSC thermograms of the PEC-ALD/CS and PEC-ALD/CS/PPy-NPs hydrogels.

In order to provide information about the physical properties (*i.e.* crystalline or amorphous nature, and the possible interactions between biopolymers) of the PEC-ALD/CS and PEC-ALD/CS/PPy-NPs gels, DSC measurements were performed.^78^ DSC thermograms of the PEC-ALD/CS and PEC-ALD/CS/PPy-NPs gels are shown in Fig. 7. Two endothermic peaks were observed for both gels. The endothermic peak between −30 and 50 °C corresponds to the melting phenomenon ascribable to water.^79^

The second detected endotherm peak at temperatures higher than 100 °C is attributed to the evaporation of water tightly linked through polar interactions to ionic groups and can be assigned to the excision of an ion pair between the carboxylic group (–COOH) of the PEC and the ammonium group (–NH_3_^+^) of the CS.^80,81^ Additionally this peak can be attributed to the decomposition of PEC-ALD (105 °C for PEC-ALD/CS/PPy-NPs gel and 115 °C for PEC-ALD/CS gel) and chitosan (145 °C for both gels).^82^

### Cyclic voltammetry

**Fig. 8:**
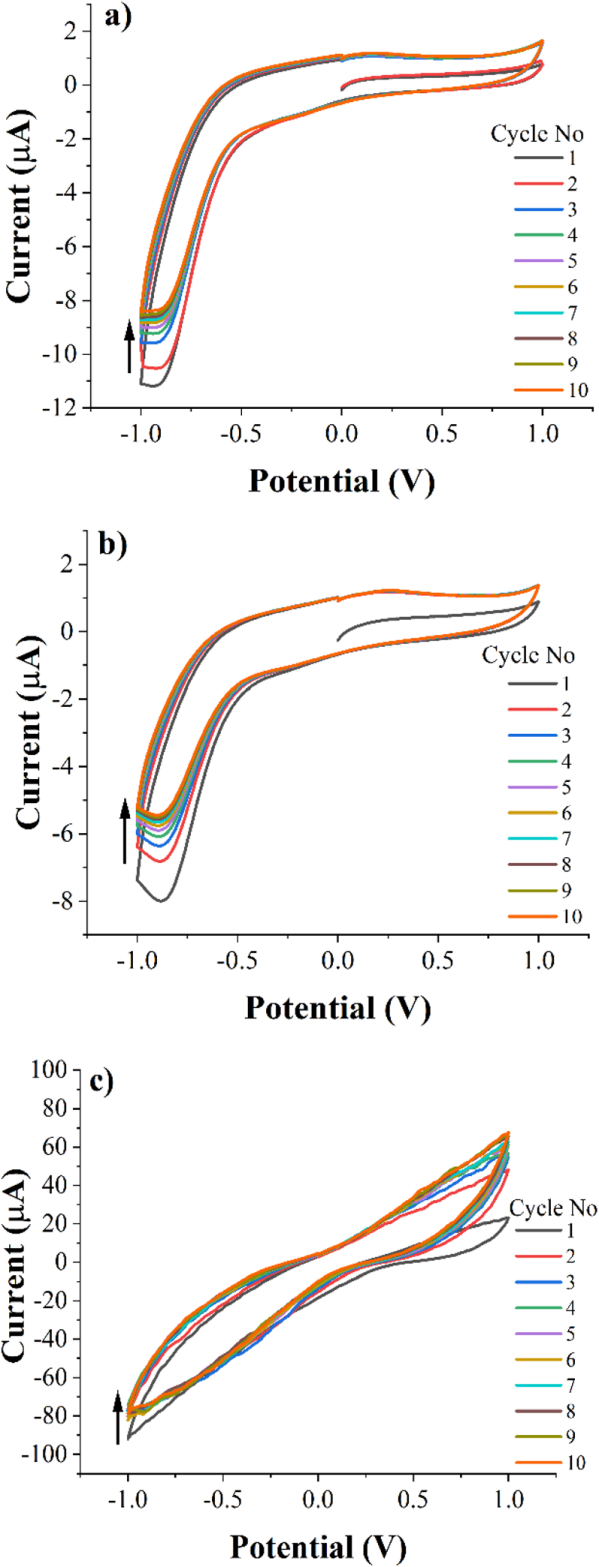
Cyclic voltammetry (CV) data of the bare GCE (a), PEC-ALD/CS *in situ* crosslinked hydrogel (b) and PEC-ALD/CS/PPy-NPs *in situ* crosslinked hydrogel (c) in phosphate-buffered saline (PBS; pH = 7.4) at a scan rate of 50 mV s^−1^.

The electrochemical properties of the PEC-ALD/CS and PEC-ALD/CS/PPy-NPs gels were studied *via* cyclic voltammetry. Fig. 8 shows cyclic voltammograms of bare GCE (a), PEC-ALD/CS *in situ* crosslinking hydrogel (b) and PEC-ALD/CS/PPy-NPs *in situ* crosslinking hydrogel (c) (1st to 10th cycles). As evident from the CV curves, the charge-storage capacities of the hydrogels decreased steadily on repeated cycling, and the areas under the curves of the 1st and 10th cycles were almost the same. The currents evolved in the PEC-ALD/CS/PPy-NPs gel were higher than those in the PEC-ALD/CS gel, and the oxidation and reduction peaks were more prominent. Occurrences of reduction and oxidation peaks correspond to the de-doping of the hydrogel (*i.e.*, release of drug molecules), and the hydrogels were subsequently re-doped by other anions (either the anionic drug, or anions from the PBS buffer: H_2_PO_4_^−^ and HPO_4_^2−^, and Cl^−^).

### Sr^2+^ release studies

**Fig. 9:**
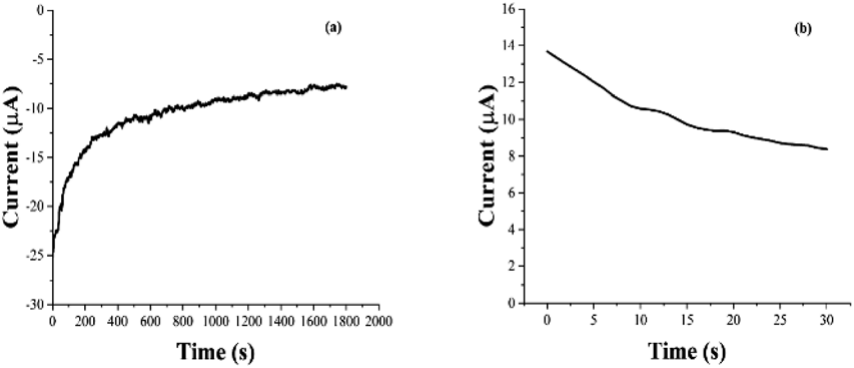
Electrochemically loading Sr^2+^ ions (a) and electrochemically enhanced delivery (b) of Sr^2+^ ions from PEC-ALD/CS/PPy-NPs *in situ* crosslinked hydrogels in PBS (pH = 7.4).

**Fig. 10:**
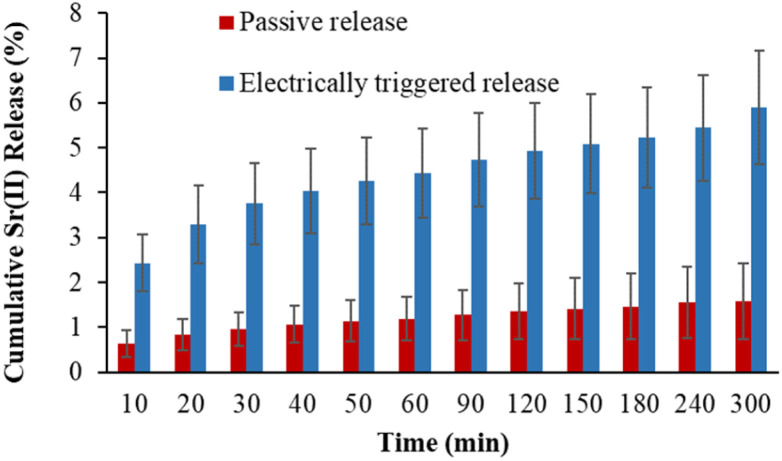
Electrochemically enhanced delivery of Sr^2+^ ions from PEC-ALD/CS/PPy-NPs *in situ* crosslinked hydrogels in PBS (pH = 7.4) as determined by ICP-OES. Cumulative release of Sr^2+^ ions from Sr^2+^ ions loaded electroactive *in situ* crosslinked hydrogels: passive release (orange bars), electrically stimulated release (blue bars).

The electrochemically triggered release of Sr^2+^ ions from PEC-ALD/CS/PPy-NPs *in situ* crosslinking hydrogels was studied using DropSens μSTAT 400 potentiostat connected to a personal computer using the Dropview 8400 software and released amount of Sr^2+^ ions were measured with ICP-OES, where the release was either passive (*i.e.*, in the absence of an electrochemical stimulus) or electrochemically triggered (*i.e.*, in the presence of one or more rounds of electrochemical stimulation: 30 s of stimulation at a reducing potential of 0.6 V, followed by 10.5 min of rest (Fig. 9)). The quantity of the released Sr^2+^ ions in solution was quantified at various time points, and the data are reported as cumulative release as a percentage of the total mass of the Sr^2+^ ions in the hydrogel (hydrogels were individually weighed; and Sr^2+^ ions concentrations of the solutions before and after Sr^2+^ ion loading were determine using ICP-OES for each hydrogel), and compared to passive drug release from unstimulated films every 11 minutes (Fig. 10).

### Antibacterial activity

**Fig. 11:**
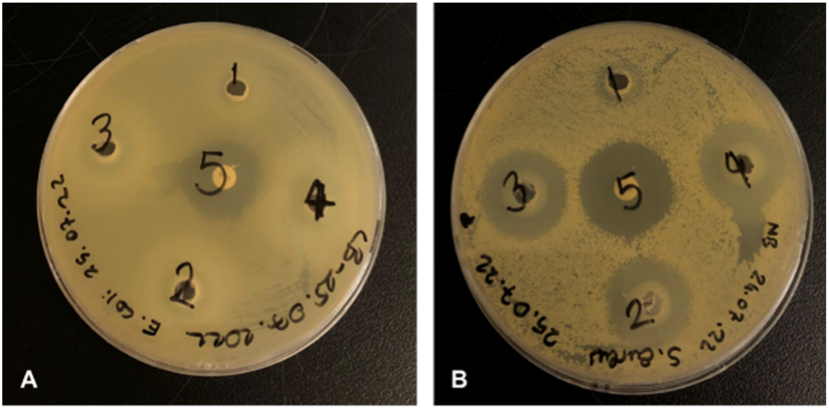
Antibacterial activities of strontium solutions at different concentrations against fresh viable *E. coli* ATCC 8739 (A) and *S. aureus* ATCC 29213 (B); 1: 10 μL (1.0 mg), 2: 50 μL (5.0 mg), 3: 75 μL (7.5 mg), 4: 100 μL (10.0 mg), 5: chloramphenicol (30 μg).

Antibacterial activity tests showed that none of the tested concentrations (1.0 mg, 5.0 mg, 7.5 mg and 10 mg) of the strontium showed antibacterial activity against *E. coli* ATCC 8739 while positive control chloramphenicol showed antibacterial activity with 20 mm inhibition zone. We found that the 7.5 and 10.0 mg strontium have antibacterial effect against *S. aureus* ATCC 29213, giving inhibition zones with values 27.8 and 28.3 mm, respectively. The inhibition zone of chloramphenicol against *S. aureus* was found to be 26 mm (Fig. 11).

**Fig. 12:**
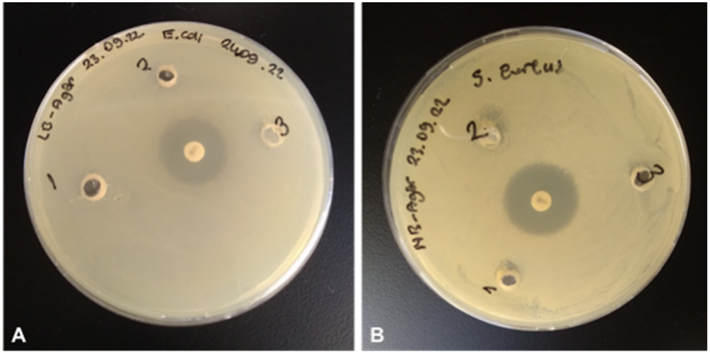
Antibacterial activities of strontium solutions electrochemically released from *in situ* crosslinking hydrogels against fresh viable *E. coli* ATCC 8739 (A) and *S. aureus* ATCC 29213 (B). (1) 39 μg mL^−1^ Sr^2+^ ions released from 100 mg hydrogel, (2) 34 μg mL^−1^ Sr^2+^ ions released from 100 mg hydrogel, (3) 31 μg mL^−1^ Sr^2+^ ions released from 100 mg hydrogel.

According to antibacterial test results, the strontium has antibacterial activity against *S. aureus* better than control antibiotic chloramphenicol while it shows no antibacterial activity against *E*. *coli.* The antibacterial properties of hydroxyapatite (HA) nanoparticles on *E. coli* and *S. aureus* were improved substituted by strontium.^83^ In another study, it was reported that strontium ranelate (SR)-loaded poly(lactic-*co*-glycolic acid) (PLGA) microspheres (PM) with assembled silver nanoparticles (AgNPs) and hydroxyapatite nanoparticles (HANPs) (SR-PM-Ag-HA) showed antibacterial activity against *Staphylococcus epidermidis* (ATCC 12228) and methicillin resistant *S*. *aureus* (MRSA; ATCC 43300), and have potential application in the treatment of bone-related infections.^84^ Different studies have shown that strontium has an antibacterial effect against *S. aureus*.^85–87^ The antibacterial activities of electrically triggered released Sr^2+^ ions from PEC-ALD/CS/PPy-NPs *in situ* crosslinking hydrogels against *E. coli* and *S. aureus* were also investigated and results are shown in Fig. 12, as can be observed the released concentrations of Sr^2+^ ions were not sufficient enough for any antibacterial activity; this observation is due to the physicochemical properties of the gels.

### Cell viability assay

**Fig. 13:**
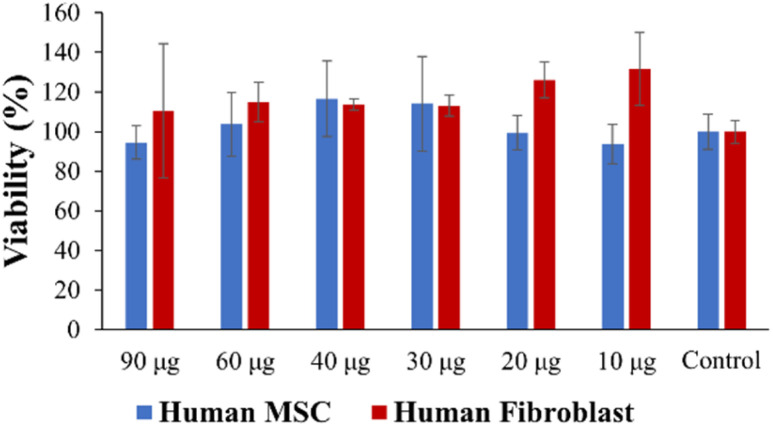
Cell viability data.

No significant toxic effects were observed in an MSC viability test *in vitro*. All fibroblast plus gel groups were found to be slightly higher in viability although there was no significant difference (Fig. 13). Therefore, the hydrogels are safe with human cells within the tested weight range (10–90 μg) *in vitro*.

## Conclusions

Herein, we report Sr^2+^ ions-loaded PEC-ALD/CS/PPy-NPs *in situ* crosslinking hydrogels which electrochemically release antibacterial Sr^2+^ ions. The gels were characterized by FTIR, SEM-EDX, TGA, DSC, XRD and CV, which confirm the successful preparation of the Sr^2+^ ions-loaded PEC-ALD/CS/PPy-NPs *in situ* crosslinking hydrogels. Thermal analysis results showed that hydrogels show good thermal stability. The swelling percentage for PEC-ALD/CS hydrogel was found to be 208 ± 10, whereas that of PEC-ALD/CS/PPy-NPs hydrogel was found to be 525 ± 7. These results are in good agreement with SEM images. PEC-ALD/CS/PPy-NPs hydrogel has larger pores while PEC-ALD/CS gels have smooth surfaces. According to CV results that demonstrating the electrochemical behaviour of the PEC-ALD/CS/PPy-NPs hydrogel, the conductivity of the hydrogels was observed to increase as the amount of PPy-NPs increases. On the other hand, addition of higher amount of PPy-NPs causes deterioration in the physical structure of the hydrogel. Due to this limitation, 5% PPy-NPs (PEC-ALD/CS/PPy-NPs-2) was decided to use for studies. The Sr^2+^ ions loaded PEC-ALD/CS/PPy-NPs *in situ* crosslinking hydrogel showed no significant cytotoxicity towards human mesenchymal stem cells (MSCs) and fibroblasts. This is the first example of Sr^2+^ loaded *in situ* crosslinking PEC-ALD/CS/PPy-NPs electroactive hydrogels that deliver the Sr^2+^ ions in a controllable manner in response to the application of electricity. The antibacterial activity of Sr^2+^ ions against *E. coli* and *S. aureus* was demonstrated and their minimum inhibitory concentrations (MIC) were determined. While these prototypical examples of Sr^2+^ loaded electroactive gels don't release sufficient Sr^2+^ ions to show antibacterial activity against *E. coli* and *S. aureus*, we believe future iterations with optimised physical properties of the gels will be capable of doing so, likewise the gels will be capable of the delivery of other divalent cations such as Ca^2+^ and Zn^2+^ ions, and therefore such *in situ* crosslinking conducting hydrogels have great promise for treatment of bone related diseases.

## Author contributions

M. F. D. conceptualized the paper and prepared the original draft. Methodology, formal analysis, and investigation were carried out by all authors. M. F. D., D. Ö. and Z. S. Ç. carried out materials and characterization. F. E. conducted antibacterial activity studies. H. D. and A. N. O. performed cell culture. M. F. D., D. Ö. and Z. S. Ç. were involved in drug delivery. M. F. D., J. G. H. and D. Ö. carried out data curation. All authors were involved in writing, reviewing, and editing. M. F. D. and J. G. H. supervised the experiments. M. F. D. led project administration and funding acquisition.

## Conflicts of interest

The authors declare no conflict of interest.

## Supplementary Material
